# Supplementary material for: Antibiotic interactions shape short-term evolution of resistance in E. faecalis
Source: PLoS Pathog. 2020 Mar 2;16(3):e1008278. doi: 10.1371/journal.ppat.1008278 (PMC7093004; doi:10.1371/journal.ppat.1008278)
Supplement: Attachment — Submitted filename: ResponseToReviews_Dean2019.pdf [file ppat.1008278.s023.pdf]

Thank you to the reviewers for the thorough reviews and suggestions, which have substantially improved the manuscript. We have addressed all reviewer concerns and hope the paper is now suitable for publication in PLOS Pathogens.

We appreciate the generally positive reviews as well as the reviewers' close attention to methodological detail. The reviewers correctly pointed out that our original manuscript was missing important details about the methodology, which led to questions about 1) the way we estimated growth and, in some cases, about 2) whether the measured differences in adaptation rate and resistance (IC50's) were statistically significant and/or dependent on the specific definitions used. The new manuscript provides additional methodological description and analysis that clarify the definition of growth used and the methods for estimating resistance. In addition, by focusing on the CRO-AMP combination—which reviewer 1 points out is the most likely to suffer from ambiguities in the estimates of growth or resistance—we provide extensive new analyses that demonstrate that 1) the adaptation rate trends do not depend sensitively on the parameters used for growth estimation or the functional form of the adaptation rate function (linear vs. saturating), and 2) the precision of our IC50 measurements is significantly better than the 2-fold differences associated with standard MIC protocols, making the changes we measure well beyond the uncertainty limitations of the measurements.

In total, the revised submission includes updated versions for 5 of 6 main text figures as well as 17 additional Supplemental Figures that address all the reviewers' suggestions in detail. We have also made minor changes to the abstract and discussion to address minor questions raised by reviewers.

In what follows, we include the original reviewer comments followed by our detailed responses (in red). We thank you again for taking the time to read, critique, and ultimately improve our work.

Reviewer #1:

I. Summary:

This is a potentially interesting study of resistance evolution to drug combinations in E. faecalis. The authors acquired growth curves of serially passaged E. faecalis in the presence of four different drug combinations, and within each combination examined each single drug and then 2 or more mixtures of the two drugs, all expected to have the same initial impact on growth rate. They conclude that adaptation trajectories are different for different dosing combinations and that this can be explained by rescaling the drug concentration to an effective concentration, by using final resistant profiles, and predicting growth rate from ancestral two drug growth response surface.

We are happy the reviewer finds the work potentially interesting.

II. Major Issues:

1) A critical missing piece to the manuscript is a description of how exactly the per capita growth rate is measured. Almost all results and analyses depend critically on this value, and there are several places where results appear to disagree with each other

The reviewer is correct. We failed to provide a thorough description of the growth rate measurement in the original manuscript, which led to ambiguities and unnecessary confusion. In the revised version, we have included more description as well as substantial new analysis of this growth rate measurement which suggests that the results do not depend sensitively on the specific parameters used for estimation. We outline these results below.

In the methods it is stated that the early exponential phase was fit to an exponential function and then normalized to ancestral cells

The growth curves of those ancestral cells are needed

Are all data normalized to one set of controls? Are they normalized to matched controls done alongside each experiment?

Why in Fig 1 and 2 do the evolved populations struggle to meet a growth rate of '1', despite acquiring resistance, whereas in Fig 3 combination D exceeds 1 – did this condition really evolve an E. faecalis strain that grows faster than wildtype?

A more precise description than "typically OD<0.4" is needed to describe how the early exponential phase of the growth curve was defined and how lag phase was avoided

Presumably the growth rate is related to the slope of the exponential portion of the OD600 over time curve. If so:
Fig 1a condition D the D0 and D3 slopes look identical, and yet the 'growth rate' is very different when plotted in Fig1b.
Fig 2a condition A, the D0 slope looks steeper than the D2 slope, and yet the 'growth rate' in Fig 2b is close to 0 for D0 and at 0.5 for D2

The above concerns are all related to measurement of growth rate, which was poorly described in the original draft. To address these issues, we did the following:

1) We now include the ancestor drug-free control growth curves in Figures 1-4. For all analyses, we now normalize growth by the rate measured in ancestral cells on the same day, which slightly changes the overall growth rates but does not impact the conclusions. This normalization more accurately accounts for day-to-day fluctuations in growth rate and eliminates some questionable numerical values (e.g. growth > 1 in Figure 3, which was due to a normalization mistake in the original draft).
2) We show the OD range (0.1-0.4) for fitting in Figures 1-4.
3) We've added the following clarification to the initial description of growth rate in the results:

Growth curves on day 0, the first day of evolution, show similar levels of inhibition for each combination, with growth initially increasing but later collapsing or plateauing. The growth curves on later days sometimes differ between replicates and between conditions (Figure 1a, right panels; Figure S1). To quantify growth, we estimated the effective growth rate during an intermediate range of OD (0.1 < OD < 0.4) for each day and each condition using nonlinear least squares fitting to an exponential function (Figure 1b). For ancestor cells in the absence of drug, this regime corresponds to exponential growth (Figure 1a, right panels; Figure S1) and this metric estimates the per capita growth rate. In cases where growth over this region is non-monotonic, this metric instead provides an effective measure of growth that decreases when population density declines, even if initial growth is rapid.

4) We have now included all growth curves in the SI (Figure S1, S16, S18, S20).

5) We have added the following description to the Methods:

The OD range [0.1, 0.4] was chosen because it spans a large region of early exponential phase growth in ancestor cells grown without drug. We normalized all growth rates by the growth rate of ancestral cells in the absence of drugs performed on the same day, with one exception: ancestor growth curves for CRO-AMP and CRO-CIP were shared between the two experiments, which were performed on consecutive days. Ancestor growth rates varied slightly day-to-day, with a minimum of 0.74 and a maximum of 0.88 hr^{-1} (doubling times of 47 and 56 mins, respectively).

6) We have also performed the analysis using different fitting parameters and adaptation rate definitions, which are now included in the SI and referenced in the main text. Specifically, we say:

These qualitative results do not depend sensitively on the specific OD window used to estimate growth (Figure S2-S3) and also hold when adaptation is estimated with a nonlinear function (Figures S4-S5). As an alternate way to visualize this adaptation, we also plotted the median growth curve (on final day of adaptation) calculated across all populations exposed to the same condition. The trends from this simple analysis are consistent with those from adaptation rate calculations–specifically, growth following adaptation to the drug combos is faster than growth following adaptation to the individual drugs (Figure S18).

2) It is difficult to assess if the differences are real and significant

-Each drug combination appears to have been examined in one serial passaging experiment, sometimes for two days, sometimes for three days. Would the trajectories and resistance evolution be reproducible in a second experiment?

It is difficult to say with certainty to what extent re-doing all the experiments would lead to changes in the evolutionary trajectories. The reviewer is correct that the serial passage experiments last for 2-3 days. To minimize batch effects due to day-to-day variability, we perform all experiments for a given drug pair together during the same 3-day period using the same batches of media, etc. We typically use 24 biological replicates per condition (96 total per drug pair), and indeed we do see variability between replicates, even when they are performed side-by-side during the same period. However, there are clear trends, which gives us hope that the results have some generality. With that said, we caution against directly comparing numerical values from experiments performed on different days—for example, experiments for drug pairs AMP-STR and CRO-AMP were performed several months apart using different batches of media and drug stocks. While our experiments provide a measure of biological variability under well-controlled conditions, we cannot be certain that similar results would hold if the experiment were performed again. Our guess, however, is that the same qualitative trends would likely hold. We have added the following brief discussion of this point to the discussion:

To minimize batch effects due to day-to-day variability (due to small changes in, for example, drug stock, media composition or temperature), we perform all experiments for a given drug pair together during the same 3-day period using the same reagent batches. We do see considerable variability between evolutionary replicates, even when they are performed side-by-side during the same period, with the same batch of media, and under the same selection conditions, giving us hope that these conditions capture at least some of the complexity of the possible evolutionary outcomes. However, it is possible that additional trajectories could arise, and perhaps even dominate, if the experiments were repeated multiple times across different days or different growth conditions.

-the adaptation rate is measured by a linear fit to very non-linear data. For many conditions it seems clear that the growth rate increases and then plateaus, so the linear fit reduces the adaptation rate. Would a better measure be time to maximum growth rate?

We considered other measures of adaptation rate, but we found that each came with drawbacks. For example, time to maximum growth rate requires us to either estimate an asymptotic (maximum) growth rate or to simply take the maximum to be the maximum of the experimental measurements, a procedure which is sensitive to small noise in the growth rate measurements. In the end, we therefore decided to stick with the simple linear approximation. In the SI, we now include an example of a different analysis for CRO-AMP where we compare results from a

linear fit to results from a fit to a saturating function. Despite the differences in definition, the same qualitative trends hold (see Figures S4 and S5).

-Resistance is reported as the change in IC50, and often the level of resistance is fairly low (a two to three-fold change), and several conclusions rest on "differences" in the time to resistance or on the degree of resistance acquired. However; it seems a typical MIC assay was used to assess resistance where cultures are grown overnight in presence of drug and then OD600 measured. This usually leads to stationary cultures below the inhibitory concentrations, and therefore a steep delineation between growth and no growth, with often 2-fold differences in MIC values for day to day assay variation for a given strain and antibiotic. This makes me skeptical that small observed changes in the IC50 are significant. The dose-response curves used to calculate IC50 could also be given as supplemental figures.

Perhaps a better dynamic range and more reliable IC50 would be obtained by measuring the growth rate of the evolved populations to a series of dilutions of the single drugs, this measure might also better align with the other data.

The reviewer makes an excellent point. Typical microdilution methods measure MIC with a precision of only about 2-fold, due in part to serial dilutions of drug. In our experiments, we used a linear gradient of drug concentrations and multiple replicates, allowing us to estimate IC50 with considerably higher precision. To illustrate this point, we include all dose response curves for the CRO-AMP combination (Figures S7-S14), which the reviewer points out (below) might be particularly sensitive to these problems. The figures include the IC50 estimate plus/minus 2 standard errors (across technical replicates). We find that the relative error is typically on the order of 10 percent, and sometimes much smaller (Figure S15). In addition, we neglected to mention that we normalize IC50 for each population by a side-by-side measurement in the ancestral cells performed on the same day. We have now added the following to the Methods to describe these points:

We chose linearly spaced drug concentrations (rather than the more conventional 2-fold dilutions (65)) to increase precision of IC50 estimates. After 20 hours of growth the optical density at 600 nm (OD600) was again measured and used to create a dose response curve. To quantify drug resistance, the resulting dose response curve was fit to a Hill-like [equation given here] using nonlinear least squares fitting, where K is the half-maximal inhibitory concentration (IC50) and h is a Hill coefficient describing the steepness of the dose-response relationship. To reduce day-to-day fluctuations, control dose response curves (in replicates of 6) in ancestral cells were measured side-by-side each day with dose response curves of the adapted populations. The IC50's from these controls were used as the normalizing factor in calculating resistance relative to ancestor (i.e. log2-scaled fold change). See Figures S7-S14 for examples. Relative error (standard error of mean / mean) for IC50 is typically on the order of ten percent and often much smaller (Figure S15).

- The combination where these issues are most clearly seen is CRO-AMP. The authors state that

for the experiment in Fig 1 “the day 2 curves vary substantially between replicates and conditions”. The day 3 curves in Fig 1a all look fairly similar to my eye, especially when compared to the other figures in the paper.

We have now included all growth curves (in separate panels) in the SI. To be conservative, we have also replaced the sentence with a weaker statement and a reference to the figure:

The growth curves on later days sometimes differ between replicates and between conditions (Figure 1, Figure S1).

Secondly they state that the “adaptation is significantly faster for the two combinations (B and C) than for the single drug treatments (A and B)”. Was a test used to assess significance? If so which one?

We performed one-sided t-tests (unequal variance) for all adaptation rate comparisons and noted the results in the figure captions (Figs 1-3). In all cases, we compare the mean adaptation rate for a drug combination (conditions B and C, combined) with those for single drugs (conditions A and B, combined).

Would the data be reproducible if the experiment was repeated again on a different day?

See response to point 1, above.

In Fig 3, condition A is the same as in Fig 1, and the growth rate curve in panel b is substantially different between days (In Fig 1 it plateaus at 0.5-0.6 on day 1, in Fig 2 it plateaus at 1 on day 2). The overall adaptation rate is similar, both close to 0.2, and so is the level of resistance measured; however the difference between the adaptation rate between experiments for condition A in Fig 1 and 3 looks similar to the difference in adaptation rate within experiment between conditions A and C in Fig 1.

Thank you for your careful reading; you helped us to avoid an embarrassing mistake. We had accidentally plotted days 0 and 1 in Figure 3 but days 0 and 3 in Figure 1. We have now correctly plotted (and labeled) D3 in both figures, and the results are similar though not identical.

As discussed above, it is somewhat difficult to compare results across experiments performed on different days with different batches of media and drugs. A more detailed comparison of the growth curves (see Figures S1 and S18, top 2 rows) reveals that the growth curves in the CRO-CIP experiment are substantially noisier than those in the AMP-CRO experiments, though as the reviewer notes, the full analysis shows that both adaptation rate and CRO-resistance levels are comparable. While we are unable to identify the exact source of the noise differences, one possibility is slight changes in humidity in the temperature controlled warm room, which leads to different levels of condensation on the plate covers and therefore different levels of aberrant light scattering.

3) A major conclusion of the paper is that rescaling concentrations based on resistance profiles predicts the growth rate in the presence of the combination. This is an attractive conclusion, most robust for the TGC-CIP combination. In this case it looks as though the predicted growth rate actually maps very closely to the achieved growth rates in Fig S4. Would be useful to plot the predicted versus actual to drive this point home?

While the scaling predictions for this case quantitatively align with predictions, we chose to omit the specific quantitative comparison because, in general, we expect the rescaling analysis to provide only qualitatively accurate predictions due to the underlying assumptions and experimental limitations. We have modified the discussion to briefly discuss this point:

As a result of these limitations, the trends predicted by rescaling can only be evaluated qualitatively, though these limitations could potentially be overcome with significantly more experimental data, leading to more quantitative rescaling predictions. For example, resistance phenotyping of individual isolates from each population could provide insight into population heterogeneity during adaptation. Nevertheless, given the potential complexity of evolutionary trajectories, it is encouraging that simple rescaling arguments can qualitatively capture the coarse-grained features we measured. Future studies that aim to overcome the technical limitations of this work may be able to further evaluate quantitative agreement between specific evolutionary trajectories and the predictions of rescaling.

However based on points listed above and below the specificity of predictions and conclusions is hard to agree with for several of the other combinations
- For combination CRO-AMP why is the predicted growth rate in Fig 5 a better than 1? Is a difference between a growth rate of 1 and 1.1 really real and significant? My conclusion would be much more general that concentration rescaling predicts in all of the combinations the adapted populations should have near wildtype growth, and that matches what was observed.

We have modified the results and clarified this analysis to reflect this suggestion:

In the CRO-AMP combination, this rescaling approach predicts that growth is slightly (approximately 10 percent or less) higher when the drugs are combined (e.g. conditions B and C) than when they are used individually (Figure 5a, top panel), in qualitative agreement with our experiments (Figure 1). More generally, the rescaling suggests that adaptation in all conditions should lead to dramatically increased growth, a consequence of the steep dose-dependence of the synergistic response surface.

- For AMP-STR and CRO-CIP the authors claim the resistant profiles fall on a line segment. For AMP-STR the resistant changes are so small that it is hard to agree with this and for CRO-CIP it actually looks more like a curve, which is what I would expect from the curved antagonistic growth contours in Fig 3 a.
The line segment analysis is unsatisfying. What was used to determine the end points X and Y of

the line segments? Why do they not lie at the x and y intercepts of the dotted lines through 0 resistance, or why do they not lie at the points of A and D, the single drug conditions?

Thank you for these points. We did not intend to over-emphasize the line segment itself. Instead, our goal was to parameterize the contour along which the "average" mutants lie and then examine different rescaling transformations along that contour. We have modified the analysis and results to de-emphasize the specific line segment, and for CRO-CIP, we indeed choose a curved (quadratic) contour to more accurately capture its shape. In addition to the new figure, we have also added the following in results:

The resistance profiles on the final day of adaptation fall at different points in the two-dimensional space describing resistance to each drug (Figure 6a and b, left panels). When profiles arising from adaptation to the same condition are averaged together, the resulting profiles (large circles) fall approximately on a smooth contour. We set out to determine how profiles at different points along these contours would be expected–based on rescaling–to impact growth in each of the four selecting conditions used experimentally.

To approximate these contours, we used a line segment (for AMP-STR) or a quadratic contour (for CRO-CIP) that connects conditions with extreme resistance. In both cases, the contours approximately run between red and blue points, corresponding to adaptation in the single drug conditions.

III. Minor Issues:

4) There are several mistakes in the manuscript, pg 3 bottom line the authors refer to day 2 curves in Fig 1 but in Fig1a the curves are labeled D3.

We have made this correction.

In Fig 1b the average rate of growth adaptation point for D has tiny error bars on it, but I do not see error bars on any other such point.

We have added error bars (+/- 1 SEM) to all such points and noted them in the captions.

On Pg 5 the authors refer to single-drug conditions (A and B), but presumably mean A and D, and this is repeated on pg 6

We have made this correction.

5) Two of the antagonistic combinations examined, AMP-STR, and CRO-CIP, include drugs that the E. faecalis strain used is already resistant to. This makes the concentrations used to study streptomycin and ceftriaxone relatively high, and lessens the relevance of the study

We have now noted this limitation in the discussion.

6) In Fig 2 parts a and b show results up to day 2, but part c resistance profiles are shown up to day 3. The data should be consistent

We made the following clarification in Results:

Note that resistance measured at the end of day 0 is considered to be resistance on day 1, as it is the expected resistance of the population at the start of day 1.

7) In the discussion the authors speculate that OD measurements at shorter intervals would allow better growth rate estimates – it is hard to imagine that acquiring more frequently than once in 20 minutes would make a drastic difference.

Good point. We have omitted this statement.

8) The growth rate curve in Fig 1b for condition D is surprising given the population is already resistant to AMP by day 1 according to Fig 1c. I suspect something went awry experimentally or with the analysis of day 1 growth rate.

We note this peculiar finding in the Results. One explanation is that the IC50 measurements may reflect the single most resistant subpopulation—not the dominant subpopulation by size. It's therefore possible that the resistance measured on day 1 reflects only a very small subpopulation, and the overall population may still appear to collapse.

We note in passing that the growth of the population in condition D is near 0 on day 1, even though resistance to ampicillin is already seen in the IC50 measurements; this peculiar finding may suggest that the population is heterogeneous, with resistant subpopulations comprising only a small fraction of the population on day 1.

Reviewer #2:

I. Summary:

Dean et al present a compelling study of how different antibiotic combination treatments affect the subsequent phenotypes evolved under such treatment. The choice of combinations is clever, demonstrating examples of synergistic and antagonistic stresses between the antibiotics. Most interesting is the tagacycline/ciprofloxacin combination that has a critical value of tagacycline concentration when there is a sudden loss of cipro resistance. Subsequently, they come up with a rescaling to normalize the various cultures to simulated constant growth. The result is quite striking: bi-antibiotic resistance profiles are essentially linear. This is a wonderful result,

showing that the phenomenological result of experimental evolution follows a quite simple rule!

We appreciate the reviewer's enthusiasm for the findings and thank him (Prof Ray) for the positive comments.

I have the following comments:

p. 6, Fig 1c: I am trying to understand how the adaptation rate can differ but the resistance is almost identical between conditions. The authors note that this is a surprising result, but I would appreciate slightly more exposition on why I should not feel so naive being surprised about it.

Our initial surprise was due to the fact that resistance (measured by MIC or IC50) is often implicitly assumed to be closely related to growth rate in the presence of drug. But indeed they are two different measures that need not be perfectly related. MIC is a measure of how much drug a population can survive, and while this may often be correlated with growth rate at a fixed concentration, it need not always be so. For example, fitness costs (lowered growth in the absence of drug) could disrupt this correlation, so the reviewer is not necessarily naïve at all. To avoid confusion, we have removed "surprisingly" and have now noted in the discussion that our study does not incorporate fitness effects.

p. 6, note on intro paragraph to section titled: "Aminoglycoside/β-lactam and β-lactam/fluoroquinolone combinations slow growth adaptation and select for resistant profiles distinct from those evolved to the component drugs." - some may note that in most papers this section would belong in the introduction, but I believe that it is well-suited here because there are multiple antibiotic combinations done, so this reminds the reader of the importance of this particular combination.

Thank you for your comment. We have elected to keep the section in the Results because, as the reviewer states, this placement allows us to reiterate the importance of the combination. But we're happy to reconsider if the editors feel strongly one way or another.

Rescaling - I personally find this to be compelling and interesting, but I fear that some in the pathogens community will not appreciate how important it is, based on my prior experience. My hope is that somewhat challenging concepts from other fields can be allowed to stand.

Thank you for your comment. We're glad you found the rescaling compelling and hope others will as well.

II. Major Issues:

No new experiments required.

III. Minor Issues:

See summary in Part I.

<u>Reviewer #3</u>:

I. Summary

In this manuscript the authors study the effect of drug combinations on the evolution of resistance. They use test cases of different drug interactions to show how it drives the resistance evolution. They use the picture of geometric rescaling to explain the results of the evolution experiments. To my knowledge, this kind of detailed experiments and analysis is innovative and can help with the optimization problem of effectiveness vs resistance. Following some revisions/clarifications, I think the manuscript will be more than adequate for publication.

We are very happy that the review finds the work innovative and suitable for publication following clarifications and revisions, which we address below.

II. Major Issues

To my opinion there are no new experiments required, but part the analysis must be revised/explained.

Looking at Figure 1a (right) the OD curves (D3) of A and D conditions looks different (it looks like in condition A the adaptation is faster), but A and D has the same adaptation rate in Figure 1b.

Similar issue is with conditions A and D in Figure 2 - it looks like the adaptation is faster condition D, but the adaptation rate are equal in Figure 2b. Moreover, it looks like the growth on day zero are not equal for all conditions.

I guess this due to the criterion for the time interval of "early exponential phase". You mentioned that it is usually OD<0.4, please elaborate more on the exact definition and the its motivation. How does the results of the paper depend on the definition? Does the main results of the paper hold with a different definition?

You mentioned this issue as a limitation: "It is clear that all growth curves are not purely exponential, and in fact the per capita growth rate can change with time. Our growth rate estimates should therefore be thought of as an effective growth rate that reduces the population dynamics each day to a single number. "

It is great that you are aware of this, but the current figures are confusing for the reader because of this issue. Additionally to a more detailed explanation of this issue in the methods section, please add the time interval that was used to estimate the growth rate in the OD figures (figure 1a,2a,...).

These issues are addressed in response to reviewer 1, which we copy below. Thank you for raising these points, which have now been clarified.

Response to Reviewer 1:

The above concerns are all related to measurement of growth rate, which was poorly described in the original draft. To address these issues, we did the following:

1) We now include the ancestor drug-free control growth curves in Figures 1-4. For all analyses, we now normalize growth by the rate measured in ancestral cells on the same day, which slightly changes the overall growth rates but does not impact the conclusions. This normalization more accurately accounts for day-to-day fluctuations in growth rate and eliminates some questionable numerical values (e.g. growth > 1 in Figure 3, which was due to a normalization mistake in the original draft).
2) We show the OD range (0.1-0.4) for fitting in Figures 1-4.
3) We've added the following clarification to the initial description of growth rate in the results:

Growth curves on day 0, the first day of evolution, show similar levels of inhibition for each combination, with growth initially increasing but later collapsing or plateauing. By contrast, the day 3 curves vary substantially between replicates and between conditions (Figure 1a, right panels; Figure S1). To quantify growth, we estimated the effective growth rate during an intermediate range of OD (0.1 < OD < 0.4) for each day and each condition using nonlinear least squares fitting to an exponential function (Figure 1b). For ancestor cells in the absence of drug, this regime corresponds to exponential growth (Figure 1a, right panels; Figure S1) and this metric estimates the per capita growth rate. In cases where growth over this region is non-monotonic, this metric instead provides an effective measure of growth that decreases when population density declines, even if initial growth is rapid.

4) We have now included all growth curves in the SI (Figure S1, S16, S18, S20).

5) We have added the following description to the Methods:

The OD range [0.1, 0.4] was chosen because it spans a large region of early exponential phase growth in ancestor cells grown without drug. We normalized all growth rates by the growth

rate of ancestral cells in the absence of drugs performed on the same day, with one exception: ancestor growth curves for CRO-AMP and CRO-CIP were shared between the two experiments, which were performed on consecutive days. Ancestor growth rates varied slightly day-to-day, with a minimum of 0.74 and a maximum of 0.88 hr−1 (doubling times of 47 and 56 mins, respectively).

6) We have also performed the analysis using different fitting parameters and adaptation rate definitions, which are now included in the SI and referenced in the main text. Specifically, we say:

These qualitative results do not depend sensitively on the specific OD window used to estimate growth (Figure S2-S3) and also hold when adaptation is estimated with a nonlinear function (Figures S4-S5). As an alternate way to visualize this adaptation, we also plotted the median growth curve (on final day of adaptation) calculated across all populations exposed to the same condition. The trends from this simple analysis are consistent with those from adaptation rate calculations–specifically, growth following adaptation to the drug combos is faster than growth following adaptation to the individual drugs (Figure S18).

III. Minor Issues

In Figure 1a, the legend is D3, in the text you mentioned day 2.

We have made this correction.

Are the OD curves in log scale? please give some indication on the figure.

The new figures have clarified this issue (yes, they are on a log scale).

Why the D0 curves doesn't start at t=0?

The drug was added at t=0 but growth is not detectable until much later on day 0.
